# The impact of previous live births on peripheral and uterine natural killer cells in patients with recurrent miscarriage

**DOI:** 10.1186/s12958-019-0514-7

**Published:** 2019-08-31

**Authors:** B. Toth, K. Vomstein, R. Togawa, B. Böttcher, H. Hudalla, Th. Strowitzki, V. Daniel, R. J. Kuon

**Affiliations:** 1 Department of Gynecological Endocrinology and Reproductive Medicine, Medical University Innsbruck, Anichstrasse 35, 6020 Innsbruck, Austria; 2 Department of Gynecological Endocrinology and Fertility Disorders, Ruprecht-Karls University Heidelberg, Im Neuenheimer Feld 440, 69120 Heidelberg, Germany; 3 Department of Neonatology, Heidelberg University Children’s Hospital, Im Neuenheimer Feld 430, 69120 Heidelberg, Germany; 4 Transplantation-Immunology, Institute of Immunology, Ruprecht-Karls University Heidelberg, Im Neuenheimer Feld 672, 69120 Heidelberg, Germany

**Keywords:** Natural killer cells, Recurrent miscarriage, Immune status, Endometrium, Immunolog

## Abstract

**Background:**

Peripheral and uterine natural killer cells (pNK and uNK cells) are key players in the establishment and maintenance of pregnancy and are disturbed in patients with recurrent miscarriage (RM). Different immunologic risk factors have been proposed between patients with primary RM (pRM, no previous live birth) and secondary RM (sRM, ≥ 1 previous live birth). However, so far, the study populations mainly consisted of small subgroups. Therefore, we aimed to analyse pNK and uNK cells in a large, well defined study population within a prospective study.

**Methods:**

In total, *n* = 575 RM patients (*n* = 393 pRM, *n* = 182 sRM) were screened according to a standard protocol for established risk factors as well as pNK and uNK cells. Peripheral blood levels of CD45^+^CD3^−^CD56^+^CD16^+^ NK cells were determined by flow cytometry and uterine CD56^+^ NK cells by immunohistochemistry in mid-luteal non-pregnant RM patients. Exclusion of patients with ≥1 established risk factor revealed *n* = 248 idiopathic RM patients (iRM, *n* = 167 primary iRM (ipRM), *n* = 81 secondary iRM (isRM)).

**Results:**

Patients with pRM and ipRM showed significant higher absolute numbers and percentages of pNK cells compared to sRM and isRM patients (pRM/ipRM vs sRM/isRM, mean ± SD /μl: 239.1 ± 118.7/244.9 ± 112.9 vs 205.1 ± 107.9/206.0 ± 105.6, *p* = 0.004/ *p* = 0.009; mean ± SD %: 12.4 ± 5.5/12.8 ± 5.4 vs 11.1 ± 4.6/11.1 ± 4.3, *p* = 0.001; *p* = 0.002). Only patients with isRM showed significantly higher uNK levels compared to patients with ipRM (mean ± SD /mm^2^ 288.4 ± 239.3 vs 218.2 ± 184.5, *p* = 0.044).

**Conclusions:**

The demonstrated differences in pNK and uNK cells in RM patients depending on previous live birth might indicate differences in NK cell recruitment and potentially different underlying immune disorders between pRM and sRM. As there is an overlap in the distribution of the NK cell results, further studies with focus on NK cell function are needed in order to clearly identify RM patients with distinct immune abnormalities. The clinical relevance of our findings should be interpreted cautiously until specificity and sensitivity are further evaluated.

## Background

Recurrent miscarriage (RM) can be differentiated in primary and secondary RM: primary RM (pRM) refers to patients with no successful pregnancy whereas secondary RM (sRM) refers to patients with at least one live birth before the miscarriages. Standardized protocols for exclusion of established risk factors in RM do not make this clinical distinction despite differences in immune regulation [1] and responsiveness towards leucocyte immunization [2] between pRM and sRM patients. As only half of RM patients can be offered targeted treatment (in about 50% of patients the cause for RM cannot be identified) we need to rethink our clinical categorization.

Natural killer (NK) cells are promising new risk factors in RM and belong to the innate immune system. They are characterized by the expression of the surface marker CD56 [3]. There are two populations that can be distinguished: peripheral NK and uterine NK cells (pNK and uNK cells). PNK cells show a strong cytotoxic activity with known antiviral and antineoplastic effects and are phenotypically and functionally different from uNK cells. UNK cells are less cytotoxic and have a different profile of secreted cytokines and receptor/gene expression, such as killer cell immunoglobulin-like receptor (KIR), human leukocyte antigen-C (HLA-C), HLA-E, and HLA-G. Both, pNK and uNK cells possess immunomodulatory functions [4, 5]. While some studies analyse NK cells as percentages of total lymphocytes, others report absolute numbers [4]. As differences were shown in percentages as well as in absolute numbers, it is advised to analyse both [4]. Ranges of NK cells are reported to vary widely and pNK cells are not routinely measured in clinical conditions other than leukaemia or transplantation immunology [6, 7]. Considering uNK cells, there have been mainly two different suggestions on references ranges, differing in the used technique of NK cell analysis. Our group focused on the number of uNK cells per mm^2^ and regarded > 300 uNK cells per mm^2^ as elevated, whereas Chen et al. focused on the percentage of total stroma cells and considered 4.5% as the upper limit of the reference range [8, 9].

UNK cells play an important role in trophoblast invasion and angiogenesis and represent about 70% of immune cells at the feto-maternal interface [10]. The exact mechanism how alterations of NK cells interfere with the development of a successful pregnancy is still a matter of debate [7, 11]. In vitro studies on vessel models have shown uNK cells leading to a disruption of vascular smooth muscle cells, which might lead to an altered spiral artery remodeling and contribute to pregnancy complications such as RM [12, 13]. In an animal model, mice lacking uNK cells, spiral artery remodeling was impaired, but was restored by transplantation of bone marrow from donors (with reconstitution of NK cells) [14–16]. Altered levels of NK cells were reported in peripheral blood, endometrium and the decidua of RM patients [4, 5]. Increased uNK cells in RM patients were described in several studies [17–19], although others could not support these findings [20, 21]. Conflicting results have also been described for pNK cells in RM patients [22, 23]. Analysing *n* = 85 RM patients and *n* = 27 controls by flow cytometry (FACS), Wang et al. did not find significant differences in pNK cell numbers [23]. In contrast, a later study reported significant higher pNK cell percentages in RM patients (*n* = 104) compared to controls (*n* = 33) [22].

A higher pNK cell number in RM patients (*n* = 210) compared to controls (*n* = 45) has been found in a retrospective study, whereas no difference in pNK cell numbers between pRM (*n* = 145) and sRM (*n* = 65) was evident [24]. In another study, a higher proportion and concentration of pNK cells in pRM compared to sRM patients was demonstrated [25]. When compared to controls, sRM patients showed higher pNK cells (absolute numbers and percentages), but the difference did not reach significance [25]. No significant differences were present with regard to uNK cells in pRM versus sRM in a small study group (pRM vs sRM: *n* = 11 vs *n* = 9) [21].

Recently, we demonstrated higher absolute pNK cells, but no differences in uNK cell levels in patients with pRM compared to sRM [1]. However, due to the limited sample size of *n* = 151 pRM and *n* = 85 sRM patients, the study was underpowered to analyse subgroups of idiopathic RM (iRM) patients. Regarding uNK cells, we were able to show elevated uNK cells in iRM patients compared to fertile controls [1].

While several studies have proposed a different immune regulation in pRM and sRM patients, sample sizes were too small to reveal differences in pNK as well as uNK cells after ruling out all established risk factors. To further delineate possibly different immunoregulatory processes between idiopathic pRM (ipRM) and sRM (isRM) patients, we analysed pNK and uNK cells in a well-defined, large cohort of RM patients in a prospective study.

## Material and methods

### Study population

Within our outpatient clinic *n* = 773 couples with RM were recruited between March 2012 and October 2018. Non-pregnant RM patients were routinely screened (RM screening test) for (i) anatomical disorders by vaginal ultrasound and office hysteroscopy; (ii) endocrine dysfunctions [polycystic ovary syndrome according to Rotterdam criteria [26], hyperprolactinemia, hyperandrogenaemia, insufficiency of the corpus luteum and thyroidal dysfunctions (hypo−/ hyperthyroidism, thyroid autoantibodies)]; (iii) autoimmune disorders (antinuclear antibodies > 1:160, anticardiolipin antibodies (IgG ≥ 10 U/ml, IgM ≥ 5 U/ml), anti-β2-glycoprotein (IgG ≥ 10 U/ml, IgM ≥ 10 U/ml), or lupus anticoagulant); (iv) deficiencies in coagulation factors (protein C, protein S, factor XII, or antithrombin); (v) inherited thrombophilia (mutations in the factor V or prothrombin gene) and (vi) parental chromosomal disorders (structural aberrations). Analyses were performed at least 3 months after the last pregnancy.

We identified *n* = 575 couples with ≥3 consecutive RM. Subgroups consisted of *n* = 393 primary RM (pRM, women who had no live births), *n* = 182 secondary RM (sRM, women who had one or more previous live births followed by ≥3 consecutive RM). After routine screening for the above-mentioned risk factors, *n* = 248 idiopathic RM (iRM) were identified, including *n* = 167 primary iRM (ipRM) and *n* = 81 secondary iRM (isRM) patients.

Diagnostics were performed in the mid-luteal phase of the menstrual cycle between day 7 and day 10 after the mid-cycle LH (luteinizing hormone) surge. Patients were advised to measure LH surge at home. Age, gravidity, body mass index (BMI), period of time since miscarriage (months), progesterone (ng/ml), thyroid-stimulating hormone (TSH, mU/l), antinuclear and thyroid autoantibodies (TPO (thyroid peroxidase antibody), thyroglobulin antibody (TG)) were evaluated as potential variables influencing NK cell number. Differences between pNK cells (per μl and percentages), uNK cells (absolute numbers per mm^2^) and correlations of these cells between the RM and iRM subgroups were defined as primary outcome measures. The analysis of variables (immune and clinical parameters) influencing pNK and uNK cells were secondary outcome measures. Characteristics of RM patients and subgroups are shown in Table 1. Signed informed consent was obtained from all participants, allowing analysis of all clinical and laboratory data mentioned in this paper.

**Table 1:** Characteristics of RM patients

	RM (*n* = 575)	pRM(*n* = 393)	sRM (*n* = 182)	*p*-value
Age^a^	34.5 ± 4.5	34.1 ± 4.7	35.3 ± 3.8	**0.0037**
Gravidity^b^	4 (3/15)	3 (3/14)	3 (3/15)	**< 0.001**
Parity^b^	0 (0/4)	0	1 (1/4)	**< 0.001**
No. of miscarriages^b^	3 (3/14)	3 (3/14)	3 (3/14)	0.68
Time since last miscarriage^a^	6.8 ± 8.9	5.7 ± 5.1	6.0 ± 5.5	0.42
BMI^a^	24.3 ± 4.2	23.98 ± 4.1	24.82 ± 4.4	0.135
P4^a^	10.6 ± 5.7	10.7 ± 5.6	10.3 ± 5.8	0.50
	iRM (*n* = 248)	ipRM (*n* = 167)	isRM (*n* = 81)	*p*-value
Age^a^	34.2 ± 4.9	33.6 ± 5.1	35.3 ± 4.2	**0.0097**
Gravidity^b^	4 (4/15)	3 (3/10)	4 (4/15)	**< 0.001**
Parity^b^	0 (0/4)	0	1 (1/4)	**< 0.001**
No. of miscarriages^b^	3 (3/14)	3 (3/10)	3 (3/14)	0.89
Time since last miscarriage^a^	6.1 ± 6.6	6.2 ± 7.3	5.8 ± 5.1	0.67
BMI^a^	24.12 ± 4.5	23.25 ± 3.9	25.4 ± 5.1	**0.029**
P4^a^	11.1 ± 6.0	11.1 ± 6.2	11.0 ± 5.7	0.90

^a^mean ± SD

^b^median (min/max)

Characteristics: Age (years), Gravidity, Parity, No. of miscarriages (number of miscarriages), Time since last miscarriage (at least 3 months), *BMI* (body mass index), P4 (progesterone levels, ng/ml) at time of immune diagnostics (luteal phase of the menstrual cycle); *RM* recurrent miscarriage, *pRM* primary RM, *sRM* secondary RM, *iRM* idiopathic RM, *ipRM* idiopathic primary RM, *isRM* idiopathic secondaryRM

### Ethical approval

The Human Investigation Review Board of the Ruprecht-Karls University Heidelberg approved the study (S-428/2009).

### Analysis of peripheral lymphocyte subpopulations

Peripheral blood levels of CD45^+^CD3^−^CD56^+^CD16^+^ NK cells were determined using four-color FACS. IgG2a/fluorescein isothiocyanate, IgG2a/phycoerythrin, IgG2a/allophycocyanin, and IgG2a/peridinin-chlorophyll-protein complex antibodies served as isotype controls. All antibodies were purchased from Becton Dickinson (BD)/ Pharmingen (Heidelberg, Germany; BD Multitest CD3/CD16 + 56/CD45/CD19, catalogue number 342446;). Ten microliters (μL) of a mixture of four different monoclonal antibodies conjugated with fluorescein isothiocyanate, phycoerythrin, allophycocyanin or peridinin-chlorophyll-protein complex were added to 50 μL of heparinized whole blood and incubated for 15 min at room temperature. Erythrocytes were lysed with NH_4_Cl for 15 min. The FACS was calibrated before each run using CaliBRITE beads (BD Pharmingen, Heidelberg, Germany) to ensure optimal counting.

### Detection of uterine natural killer cells

A uterine biopsy was taken in *n* = 346 patients in the mid-luteal phase using a Pipelle sampler (Pipelle® CCD, Laboratoire CCD, Paris, France) to evaluate uterine CD56^+^ NK cells by immunohistochemistry. All endometrial biopsies were fixed in 5% buffered formalin for at least 24 h and embedded in paraffin. The samples were cut at 4 μm, mounted on SuperFrost/Plus slides (Menzel, Germany) and deparaffinized and rehydrated. Antibodies were diluted with Background Reducing Components (DAKO, Germany). Antigen retrieval was accomplished by using citrate buffer. To inhibit endogenous peroxidase activity, samples were incubated with Peroxidase Block (DAKO, Germany) for 7 min as recommended and washed in TBS-Tween20 (0.05%; TBS, pH 7.6). Samples were incubated with the primary mouse anti-human CD56 antibody (clone:123C3, isotype: IgG1, DAKO, Germany, catalogue number: M730401–2, concentration: 305 mg/l, used with 1:100) for 1 h and for 30 min with the secondary antibody (labelled polymer-HRP anti-mouse, clone: DAK-GO1, isotype: IgG1, DAKO, Germany, catalogue number: K800021–1) at room temperature. Between each step, all samples were washed profusely with TBS-Tween20 (0.05%). The peroxidase reaction was achieved with DAB (3.3′-diaminobenzidine; DAKO, Germany) and discontinued with water after 15 min. Haematoxylin staining was followed by mounting the cover slide with Histofluid (Marienfeld, Germany). All samples were analysed independently by two experienced biologists/physicians using a Zeiss AxioPlan Microscope and the AxioVison 4.8 program. CD56^+^ uNK cells were evaluated as absolute numbers per mm^2^_._

### Statistics

Statistical analysis was performed using GraphPad Prism (version 7.00 for Windows, GraphPad Software, La Jolla California USA, www.graphpad.com).

In case of normally distributed raw data, student’s t-test was used to compare two groups and one-way ANOVA was used for comparison of more than two groups, followed by Holm-Sidak’s multiple comparison test. Otherwise, Kruskal-Wallis for non-parametric testing was used to compare groups followed by Dunn’s multiple comparison test. Correlations between parameters were calculated by means of Pearson’s correlations coefficient, since the analysed data were ratio-scaled. Data of dichotomous variables were compared by Chi-square test. A *p*-value < 0.05 was considered significant.

## Results

### Study population

Characteristics of RM patients are displayed in detail in Table 1. Number of miscarriages, time passed after the last miscarriage and luteal phase progesterone levels did not differ between the subgroups of RM patients. Patients with isRM had a significant higher BMI than patients with ipRM. Mean age, gravidity and parity of patients were significantly higher in sRM and isRM versus pRM and ipRM patients respectively.

### Peripheral natural killer cells

**Fig. 1:**
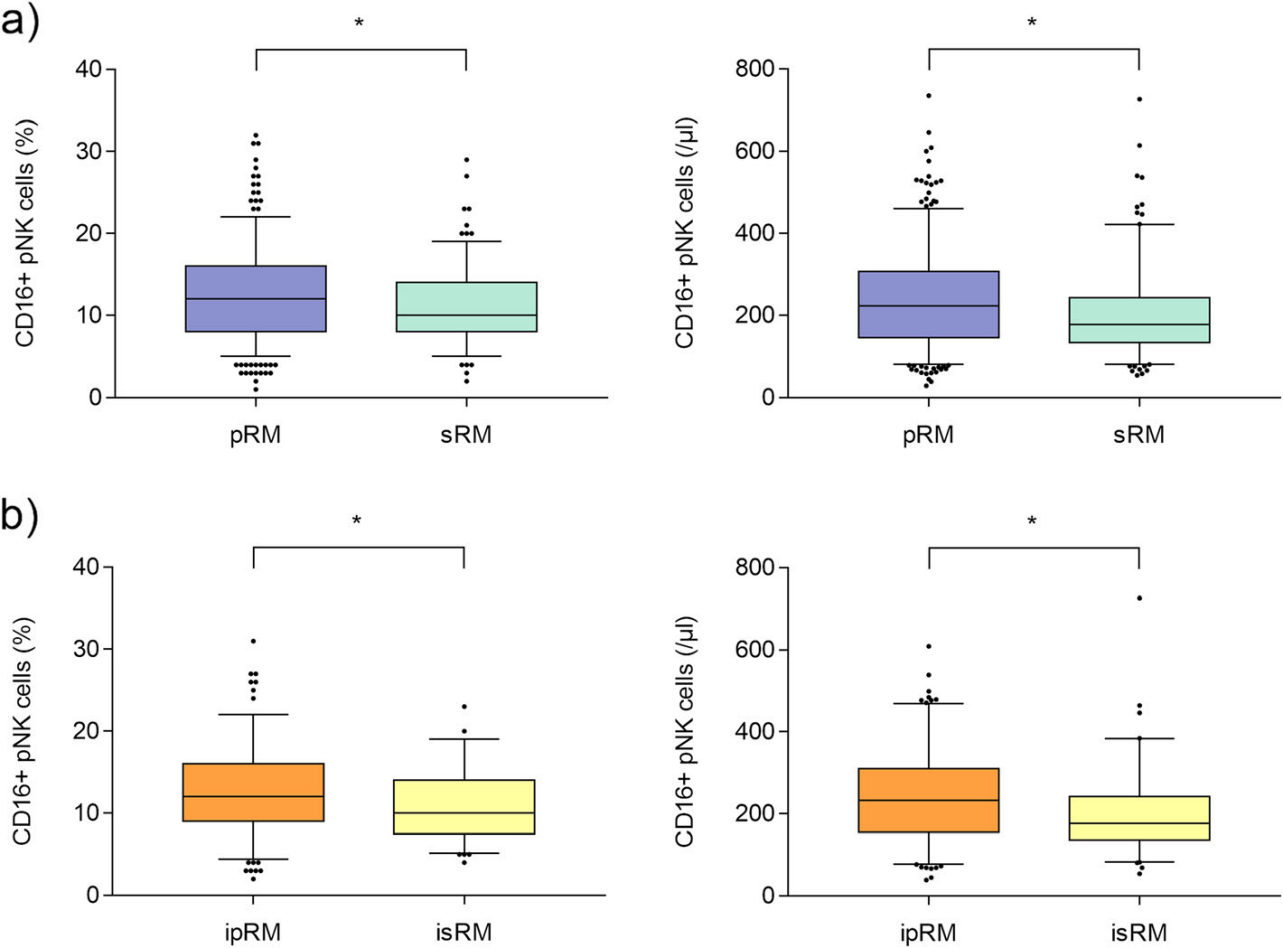
CD45^+^CD3^−^CD56^+^CD16^+^ pNK cells (percentages and absolute numbers) in RM (**a**) and iRM (**b**) patients. CD45^+^CD3^−^CD16^+^CD56^+^ pNK cells were significantly higher in (i) pRM than (i) sRM patients (percentages and absolute numbers /μl). Whiskers show 5 and 95% percentiles, *p* < 0.05 was considered significant, (i) pRM = (idiopathic) primary recurrent miscarriage, (i) sRM = (idiopathic) secondary recurrent miscarriage

Peripheral CD45^+^CD3^−^CD56^+^CD16^+^ NK cells of RM patients are shown in Table 2. As shown in Fig. 1a and b, the distribution of NK cell numbers and percentages is widespread. Compared to patients with sRM, women with pRM showed higher pNK absolute numbers and percentages (Table 2; Fig. 1a). These differences were also present in idiopathic RM patients in the corresponding subgroups (Table 2, Fig. 1b). No significant difference was observed when comparing patients with ipRM vs non-ipRM and isRM vs non-isRM.

**Table 2:** CD45^+^CD3^−^CD56^+^CD16^+^ pNK cells and CD56^+^ uNK cells in RM patients

		RM (*n* = 575)	pRM (*n* = 393)	sRM (*n* = 182)	*p-*value
CD45^+^CD3^−^CD56^+^CD16^+^ pNK cells	Percentages (mean ± SD)	12.0 ± 5.3	12.4 ± 5.5	11.1 ± 4.6	**0.0043**
	Absolute numbers (mean ± SD)	228.3 ± 116.4	239.1 ± 118.7	205.1 ± 107.9	**0.0011**
CD56^+^ uNK cells	Absolute numbers (mean ± SD)	215.2 ± 180.3 (n = 346)^a^	205.4 ± 170.2 (*n* = 245)^a^	238.8 ± 201.6 (*n* = 101)^a^	0.12
		iRM (*n* = 248)	ipRM (*n* = 167)	isRM (*n* = 81)	*p-*value
CD45^+^CD3^−^CD56^+^CD16^+^ pNK cells	Percentages (mean ± SD	12.2 ± 5.1	12.8 ± 5.4	11.1 ± 4.3	**0.0181**
	Absolute numbers (mean ± SD)	232.2 ± 111.9	244.9 ± 112.9	206.0 ± 105.6	**0.0099**
CD56^+^ uNK cells	Absolute numbers (mean ± SD)	242.5 ± 207.1 (*n* = 156)^a^	218.2 ± 184.5 (*n* = 102)^a^	288.4 ± 239.3 (*n* = 54)^a^	**0.044**

*RM* = recurrent miscarriage, *pRM* = primary RM, *sRM* = secondary RM, *iRM* = idiopathic RM, *ipRM* = idiopathic primary RM, *isRM* = idiopathic secondary RM.

^a^indicates the number of patients in which a uterine biopsy was obtained

### Uterine natural killer cells

**Fig. 2:**
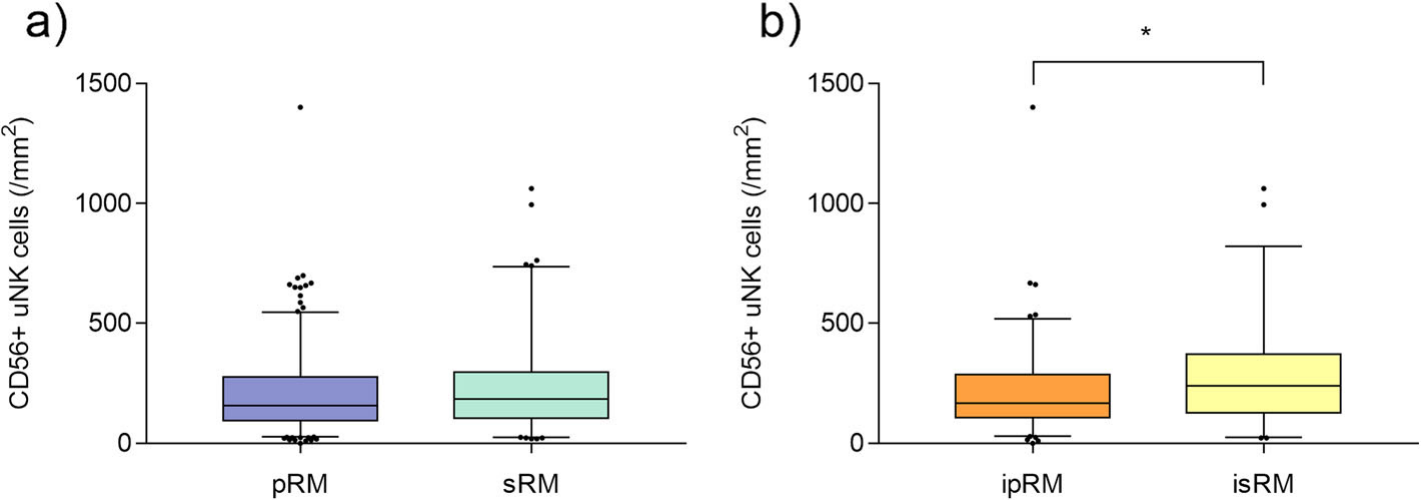
CD56^+^ uNK cells in RM (**a**) and iRM (**b**) patients. There was no significant difference of CD56^+^ uNK cells between patients with pRM and sRM. Patients with isRM showed significantly higher uNK cell levels/ mm^2^ compared to patients with ipRM. (i)PRM = (idiopathic) primary RM, (i)sRM = (idiopathic) secondary RM.

Numbers of CD56^+^ uNK cells/ mm^2^ in the different RM subgroups are shown in Table 2. There was no significant difference of uNK cells between patients with pRM and sRM. However, when focusing on idiopathic RM, patients with isRM showed significantly higher uNK cell levels/mm^2^ compared to patients with ipRM (Table 2; Fig. 2). Further, patients with isRM showed higher uNK cells than patients with non-isRM (288.4 ± 239.3 vs 180.9 ± 127.1, *p* = 0.007). No significant difference of uNK cells was detected between ipRM and non-ipRM. When put into categories of potential reference ranges that have been proposed in a previous study [9], highly elevated uNK cells (> 600/mm^2^) were more present in patients with isRM compared to patients with ipRM patients (*p* = 0.04, Table 3).

**Table 3:** CD56^+^ uNK cells in RM patients – Number of patients within previously proposed reference ranges (9)

Range of CD56^+^ uNK cells	Number (percentage) of patients within range			
	RM (*n* = 346)	pRM (*n* = 245)	sRM (*n* = 101)	*p*-value
< 40	29 (8.38)	22 (8.98)	7 (6.93)	0.53
≥40 < 300	238 (68.78)	169 (68.98)	69 (68.32)	0.90
≥300	79 (22.83)	54 (22.04)	25 (24.8)	0.58
> 600	15 (4.335)	9 (3.67)	6 (5.94)	0.30
	iRM (*n* = 156)	ipRM (*n* = 102)	isRM (*n* = 54)	*p*-value
< 40	12 (7.69)	8 (7.84)	4 (7.41)	0.92
≥40 < 300	102 (65.39)	70 (68.63)	32 (59.26)	0.24
≥300	42 (26.92)	24 (23.53)	18 (33.33)	0.18
> 600	9 (5.77)	3 (2.94)	6 (11.11)	**0.04**

*RM* recurrent miscarriage, *pRM* primary RM, *sRM* secondary RM, *iRM* idiopathic RM, *ipRM* idiopathic primary RM, *isRM* idiopathic secondary RM.

### Correlation between uterine and peripheral NK cells

We find a moderate positive correlation between CD56^+^ uNK cells and CD45^+^CD3^−^CD56^+^CD16^+^ pNK cells only in ipRM patients (/μl: r = 0.393, *n* = 102, *p* < 0.001; percentages: *r* = 0.331, *n* = 102, *p* < 0.001). In contrast, a weak negative correlation was detected between CD56^+^ uNK cells and absolute numbers of CD45^+^CD3^−^CD56^+^CD16^+^ pNK cells in isRM patients (*r* = 0.301, *n* = 54, *p* = 0.027).

## Discussion

Due to the various established risk factors, study populations of patients with RM are characterized by a distinct heterogeneity. Finding and describing new aspects of immune regulation on the one hand and confirming results from studies with smaller sample size in large populations on the other hand will lead to a better understanding of the pathophysiology of RM.

Higher absolute numbers but not percentages of pNK cells were detected in *n* = 151 patients with pRM compared to *n* = 85 patients with sRM [1]. Within the current study the distribution of pNK cell numbers (absolute and percentages) was widespread, which was also shown in other studies analysing lymphocytes by FACS [6]. Still, both absolute numbers as well as percentages of pNK cells were significantly higher in patients with pRM compared to sRM, confirming the findings of our previous study [1]. Higher activity of pNK cells was shown in pRM compared to sRM patients in a study by Shakar et al. [25], underlining a possible impact of previous live births on NK cells in sRM patients.

Due to their different phenotype and the missing detection of a correlation between pNK and uNK cells, we and others have suggested categorizing these lymphocytes as two independent immune markers for RM [1, 27]. Basic science has shown the essential role of uNK cells in successful development of the placenta, e.g. the involvement in the remodelling of the spiral arteries [12, 28, 29]. Elevations of uNK cells have been associated with hypertensive disorders of pregnancy, preeclampsia and fetal growth restriction [30–32]. Significantly higher uNK cell numbers have previously been described in patients with iRM (≥ 3 consecutive clinical miscarriages) compared to fertile controls [9]. Yet, there has been no international consensus on the standardization of uNK cell testing in RM patients. Consequently, reference ranges of low, normal and elevated uNK cells need to be established. Considering the reference ranges proposed by our group and by Chen et al., 34.5% respectively 22% of iRM patients showed elevated uNK cells and 3% respectively 16% of iRM patients low uNK cells [8, 9]. However, these two studies did not show differences between patients with ipRM and isRM. Our current study shows no significant difference in low, normal and elevated uNK cells between ipRM and isRM either. However, absolute uNK cells / mm^2^ as well as the fraction of highly elevated uNK cells (defined as > 600 uNK cells/ mm^2^) are significantly higher in patients with isRM compared to ipRM (both *p* = 0.04), stressing the need to investigate subpopulations and the impact of a previous live birth. A previous study on uterine NK cells comparing patients with primary versus secondary infertility has proposed a different immune regulation, showing higher uNK cells in secondary infertility patients [33].

We hypothesize that differences of pNK and uNK cells between pRM and sRM reflect an interaction with fetal microchimeric cells. The mechanism of feto-maternal microchimerism describes the bidirectional traffic of cells across the placenta resulting in an antigenic challenge [34]. This process starts as early as 7 weeks of gestation and therefore also in patients with pRM [35]. However, feto-maternal microchimerism reaches a maximum at delivery, which only occurs in patients with sRM [35]. Obstetric and neonatal complications are associated with an increased transfer of fetal cells into maternal circulation [36–39] and an increased production of inflammatory cytokines in the peripheral blood and endometrium [40–42]. Studies have shown a higher rate of gestational complications in sRM during their first pregnancy and delivery, indicating an increased transfer of fetal cells [43, 44]. These cells, persisting for up to 27 years, might induce a chronic immune stimulation, resulting in a disturbed immune regulation in the mother with lower absolute numbers and percentages of pNK cells [45]. This hypothesis is in line with the results of previous studies showing a decrease in pNK cells and NK cell cytotoxicity during pregnancy and postpartum, which might be a maternal response to fetal microchimeric cells [46, 47]. As feto-maternal microchimerism is a physiological process, RM patients might fail to adapt adequately to the challenge the microchimeric cells oppose on the maternal immune system causing a different immune reaction towards the newly implanting embryo with lower pNK and higher uNK cells in sRM patients.

Of note, the (i) pRM and (i) sRM groups in our study showed significant differences in age and BMI, which might confound our findings. A study of our group showed no influence of clinical parameters like BMI, age, time of last miscarriage or progesterone levels on pNK and uNK cell numbers [1]. Furthermore, the influence of body weight and age on lymphocyte counts is discussed controversially and studies did not compare slight differences in BMI and age like in our study population [48–50]. As described before, this study was not designed to study differences in pRM or sRM in comparison to controls. To study these differences, one would have to study two groups: controls that have already had a live birth and controls that never had a live birth. In general, the most appropriate control group for RM has yet to be defined.

## Conclusion

The interaction between pNK and uNK cells is a matter of debate [1, 51] and so far, no direct correlation has been shown in studies. In our large, well defined cohort of women with RM, allowing for discrimination between patients with pRM and sRM, we are the first to show a positive correlation of pNK and uNK cells in patients with ipRM. Interestingly, patients with isRM show higher uNK cells, but lower pNK cells compared to women with ipRM, indicating a possible abnormal recruitment of NK cells from peripheral blood to the endometrium. In conclusion, this study indicates that there might be a different profile of NK cells between patients with pRM and sRM. These immune alterations in pRM and sRM could contribute to a different aetiology of RM. As some findings are only evident in iRM patients, it stresses the need to exclude established risk factors for RM before immune markers like NK cells are investigated in the peripheral blood and endometrium. Due to the overlap of the distribution of NK cell results in RM patients, further studies focusing on the function of pNK and uNK cells are needed in order to clearly identify RM patients with distinct immune abnormalities. The clinical relevance should be interpreted with caution until specificity and sensitivity of these markers are further evaluated.

## Data Availability

The datasets used and/or analysed during the current study are available from the corresponding author on reasonable request.

## Abbreviations

BMI: Body mass index
HLA: human leukocyte antigen
ipRM: Primary idiopathic recurrent miscarriage
iRM: Idiopathic recurrent miscarriage
isRM: Secondary idiopathic recurrent miscarriage
LH: Luteinizing hormone
NK cells: Natural killer cells
pNK cells: Peripheral natural killer cells
pRM: Primary recurrent miscarriage
RM: Recurrent miscarriage
sRM: Secondary recurrent miscarriage
TG: thyroglobulin antibody
TPO: thyroid peroxidase antibody
TSH: thyroid-stimulating hormone
uNK cells: Uterine natural killer cells
